# Non-Destructive Testing of a Fiber-Web-Reinforced Polymethacrylimide Foam Sandwich Panel with Terahertz Time-Domain Spectroscopy

**DOI:** 10.3390/s24061715

**Published:** 2024-03-07

**Authors:** Yu Liu, Yefa Hu, Jinguang Zhang, Haixin Liu, Meng Wan

**Affiliations:** 1 School of Mechanical and Electronic Engineering, Wuhan University of Technology, Wuhan 430070, China; liuy204@126.com (Y.L.);; 2 Institute of Advanced Material and Manufacturing Technology, Wuhan University of Technology, Wuhan 430070, China; 3 Hubei Provincial Engineering Technology Research Center for Magnetic Suspension, Wuhan 430070, China

**Keywords:** non-destructive testing, terahertz, fiber-web-reinforced polymethacrylimide foam sandwich panels, imaging algorithm, super-resolution reconstruction

## Abstract

Terahertz (THz) non-destructive testing can detect internal defects in dielectric materials. However, this technology is mainly used for detecting thin and simple structures at present, lacking validations for the detection effectiveness of internal defects in thicker and more complex structures, such as fiber-web-reinforced composite sandwich panels. In this study, samples of fiber-web-reinforced polymethacrylimide foam sandwich panels, which are, respectively, 20 mm and 30 mm thick, were made to detect the internal debonding, inclusion, pore, and crack defects by the THz time-domain spectroscopy system (THz-TDS). The peak-to-peak-imaging algorithm, maximum-amplitude-imaging algorithm, minimum-amplitude-imaging algorithm, pulse-width-imaging algorithm, and time-of-flight-imaging algorithm were used to process and image the collected THz signals. The results showed that the peak-to-peak-imaging algorithm had the best performance. To address the low imaging resolution of THz-TDS, a block-based super-resolution reconstruction method—SSSRGAN—is proposed, which can improve image resolution while maintaining the clear edge contours of defects. The defect-detection results of the samples showed that THz-TDS could detect all pore, debonding, and crack defects, with a minimum size of 3 mm for pores and debonding and a minimum thickness of 1 mm for cracks. The method showed poor detection performance for inclusions with a thickness of 0.053 mm, but could still extract the defect features. Based on the THz-TDS reflection mode measurement principle, the thickness information of the panel, foam core, and web of the samples was calculated: the measurement error was no more than 0.870 mm for Sample #1 and no more than 0.270 mm for Sample #2, demonstrating the accuracy of THz-TDS in measuring the dimensions of sandwich panel structures. In general, THz technology shows potential for detecting internal defects and performing dimensional measurements in complex structures. With the advancement of portable devices and enhancements in detection speed, real-time on-site detection is anticipated in the future.

## 1. Introduction

Composite sandwich structures, composed of high-strength panels and lightweight core materials, feature light weight, high strength, and good corrosion resistance advantages and have been widely used in various industries [1,2]. For traditional composite sandwich structures, the lightweight materials with a low modulus are mainly used as the core materials, which have certain drawbacks under out-of-plane loads, such as a low modulus and large shear deformation. Meanwhile, the traditional composite sandwich structure is also prone to the brittle failures of interface delamination and core shear, which restrict the full utilization of the fiber material’s strength. In view of the above, a new type of fiber-web-reinforced composite sandwich panel (FWRCSP) has been proposed in recent years, which consists of top and bottom panels, a fiber web, and a foam core [3]. This new FWRCSP effectively improves the interface performance of traditional sandwich panels, enhances the bending and shear ultimate load capacity and stiffness, and has been widely used in engineering fields such as road mats, vehicles, ships, and high-speed trains [4,5].

However, due to the presence of the web, the area to which the foam core bonds to the panels and the web is significantly increased, making it prone to the defects of cracks, debonding, inclusions, and pores during production or application [6,7]. These defects could be gradually enlarged during use, eventually leading to the failure of the sandwich panel and posing safety hazards. Therefore, it is necessary to detect the internal defects of the sandwich panel.

Methods for detecting internal defects in composite materials include ultrasonic testing [8], infrared testing [9], eddy current testing [10], X-ray testing [11], etc. Each of the above has its own advantages and disadvantages. Terahertz (THz) non-destructive testing, as an emerging technology, can effectively penetrate non-conductive materials to detect and image the internal defects of composite materials [12,13]. It is a complementary technique to other testing methods. THz is characterized by small scattering, higher imaging resolution compared to ultrasonic waves, semi-transparency, low electronic energy, and higher safety [14]. In the testing of foam materials, strong scattering of ultrasonic waves appears due to the porous nature of the foam material, which further causes large signal attenuation. Therefore, the conventional ultrasonic testing method can hardly meet the requirements for imaging quality. Compared to ultrasonic testing, THz waves have lower energy attenuation when propagating in the medium, making them able to perform detection in deeper places of the medium. X-rays have low imaging contrast for different types of defects in foam core materials and cannot clearly distinguish defect features, while THz waves show refractivity changes at the defect location, resulting in good imaging contrast for defects. THz is also safer than X-rays [15]. Due to the good thermal insulation properties of foam materials, infrared testing is poor in detecting defects deep inside the foam, while THz waves have relatively small scattering and good penetration, making them suitable for detecting defects in thicker objects.

Currently, researchers have conducted few research works on defect detection in composite materials using THz technology. Xing et al. used THz reflection mode to test debonding and inclusion defects in polymethacrylimide (PMI) foam and image the defect areas, verifying the feasibility of using THz to detect PMI foam defects [16]. Yang et al. improved the accuracy of THz detection in composite materials using deep learning methods [17]. Balbekin et al. used a THz detection system to identify the chemical composition of spars in aircraft and rotor blades and detected pores and delamination defects in objects [18]. Xing et al. also employed THz reflection mode to detect crack and void defects in PMI foam, yielding successful detection outcomes [6]. Han et al. conducted protein analysis using THz technology, demonstrating that terahertz spectroscopy is effective in discerning different protein types and measuring protein content [19]. Okano et al. utilized THz polarization spectroscopy to analyze the internal state of opaque rubber, establishing a mathematical model relating birefringence (optical) information to strain (mechanical) information [20].

However, there is currently no research on the application of THz in detecting internal defects in thick and complex structures like foam sandwich panels. The advantages of THz, including small scattering, good penetration, and safety, guarantee great potential for using it in non-destructive testing of foam sandwich panels. In this case, it is urgently needed to conduct research in this area. In particular, the FWRCSP structure is more complex compared to traditional foam sandwich panels, for its web in the middle part results in multiple refractions and reflections of THz waves in different media layers, affecting the detection results. Therefore, in order to verify the feasibility of using THz non-destructive testing to detect FWRCSP defects, relative FWRCSP samples with different thicknesses and four prefabricated types of defects (debonding, inclusion, pore, and crack) were designed in this study. In addition, based on the THz time-domain signal, the thicknesses of the panel, foam core, and web of the testing samples were calculated, expanding the application prospects of THz non-destructive testing in sandwich panel structure measurement. The comparison of our work and the other works is shown in Table 1.

## 2. Sample Preparation

### 2.1. Materials

The FWRCSP was composed of panels, webs, and PMI foam cores. The panels and webs were made of glass-fiber-reinforced plastic (GFRP), which consists of resin and glass fiber in a composition ratio of 1:4, with a density of 1.9 g/${\mathrm{cm}}^{3}$. The resin used in the sample was the YPH-629 epoxy resin provided by Kunshan Yubo Composite Materials Co., Ltd. (Kunshan, China), and the glass fiber used in the sample was the EC600 glass fiber yarn provided by Jiangsu Jiuding New Materials Co., Ltd. (NanTong, China). The model of the PMI foam is HnBxFoam-X-110 with a density of 0.11 g/${\mathrm{cm}}^{3}$. PMI is characterized by a high specific strength, high specific modulus, high heat resistance, compression resistance, corrosion resistance, and good dielectric properties. It is an ideal core material for high-performance sandwich structure composites [21]. In contrast to conventional foam materials, employing PMI as the foam core in the FWRCSP substantially enhances the bending and shear ultimate load capacity and stiffness, thus facilitating the widespread application of the FWRCSP in aerospace and aviation industries.

### 2.2. FWRCSP Samples

**Figure 1:**
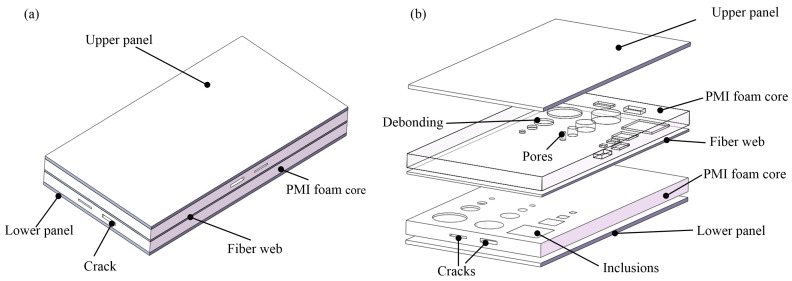
The structure of fiber-web-reinforced polymethacrylimide foam sandwich panels: (**a**) Axis side view. (**b**) Exploded view.

The FWRCSP sample was 150 mm long and 80 mm wide. The thicknesses of Sample #1 and Sample #2 were 20 mm and 30 mm, respectively. The thicknesses of the upper and lower panels were 2 mm, made of stacked GFRP fabric with a ply angle of ${\left[0/90\right]}_{5}$. The web was 1.2 mm thick with a ply angle of ${\left[0/90\right]}_{3}$. The sandwich panel structure is as shown in Figure 1.

**Figure 2:**
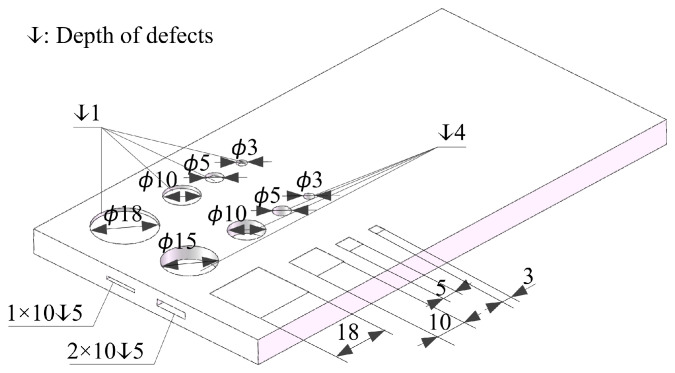
The defect size information (unit: mm).

**Figure 3:**
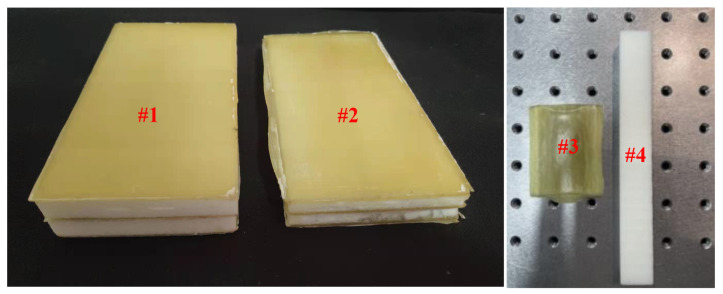
Optical images of the sandwich panel samples (Sample #1 with a thickness of 30 mm; Sample #2 with a thickness of 20 mm), GFRP laminate (#3), and PMI foam (#4).

Debonding (circular) and inclusion (square) defects were prefabricated on the bonding surface between the web and the foam. The defect sizes were 18 mm, 10 mm, 5 mm, and 3 mm. The debonding defect was simulated by filling the bonding surface with multiple layers of Teflon films, and the inclusion defect was simulated by inserting paper into the bonding surface. Due to the high stiffness of PMI foam, the potential for pore and crack defects increases during manufacturing or utilization. The detection of these defects can significantly enhance the safety of the FWRCSP. To validate the feasibility of defect detection, simulating these defects is imperative. Circular pore defects with sizes of 15 mm, 10 mm, 5 mm, and 3 mm were excavated in the upper and lower PMI foams. Cracks with widths of 1 mm and 2 mm were created on the side of the foam. The defect size information is shown in Figure 2. Optical images of the sandwich panel samples, GFRP laminate, and PMI foam are shown in Figure 3.

## 3. Method

### 3.1. THz Time-Domain Spectroscopy System

THz time-domain spectroscopy (THz-TDS) system (TeraMetrix T-Ray 5000) (Roanoke, VA, USA) was used for detection in this study. The system adopts a fully optical fiber design, with a spectral range of 0.1–3.5 THz, a sampling frequency of 100 Hz, a spectral dynamic range of ≥65 dB, and a maximum time-domain scanning range of 700 ps. It is suitable for high-precision spectral analysis.

**Figure 4:**
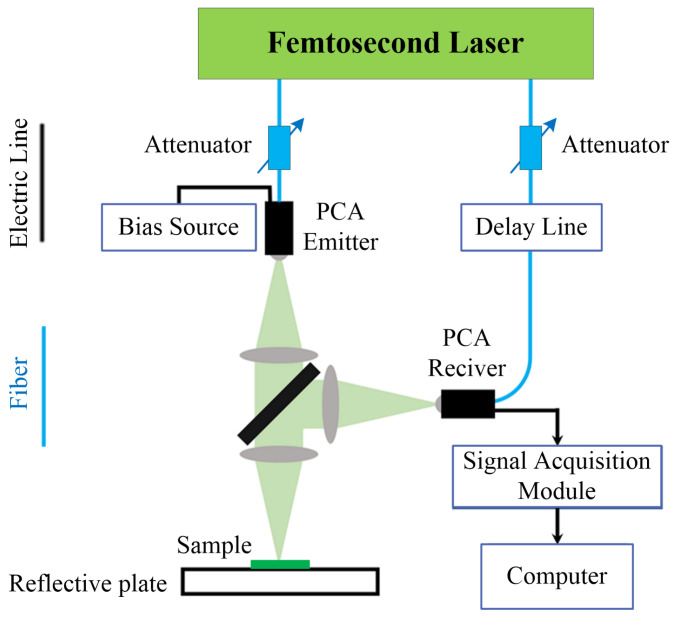
The THz-TDS reflection mode measurement principle.

**Figure 5:**
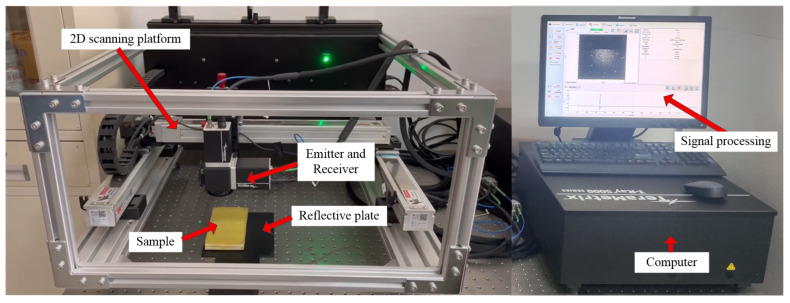
The THz-TDS system equipment.

The principle of THz-TDS is to split the femtosecond laser beam into a strong pump beam and a weak probe beam by a beam splitter. The pump beam passes through a time delay system and then reaches a THz radiation source to excite THz pulses. After focusing, the THz pulses are directed towards the sample. And the THz pulses transmitted out through sample and the probe beam together enter the THz detector simultaneously. By adjusting the time delay system, the time delay between the pump beam and the probe beam can be controlled, thus allowing the determination of the overall THz pulse waveform of the sample in the time domain. THz-TDS can be divided into transmission mode and reflection mode. Since reflection mode contains depth information, it was adopted in this study to detect internal defects in the sample. The relative principles are shown in Figure 4. And the THz-TDS system equipment is shown in Figure 5.

**Figure 6:**
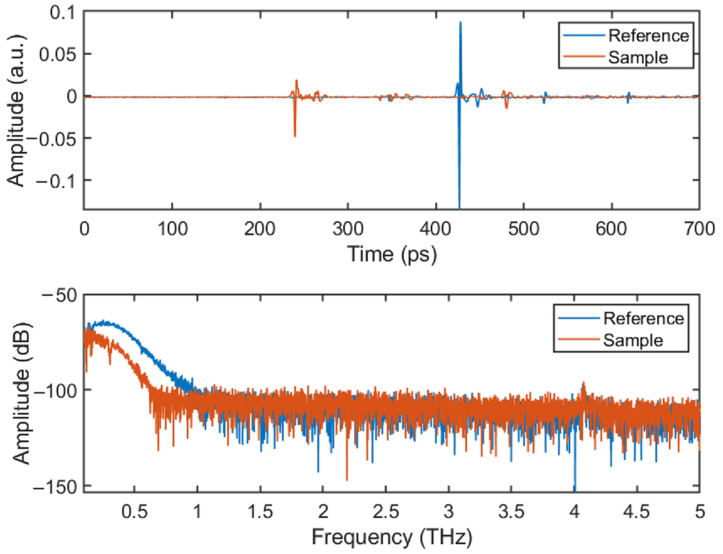
The comparative analysis (time-domain spectrogram and frequency-domain spectrogram) between the reference signal and sample signal.

The THz-TDS reflection mode was used to test the samples. And a comparative analysis was conducted between the reference signal and sample signal; please see Figure 6.

It can be seen from the time-domain spectrogram that the reference signal had a significant peak at 420 ps. This is because the THz wave propagates from the air (low refractive index) to the metal plate (high refractive index), resulting in a significant reflection waveform due to the large difference in the refractive index (which also leads to a large difference in the reflection coefficients). Similarly, the sample signal also had significant peaks at 235 ps and 420 ps. The peak at 235 ps represents the THz wave propagating from the air to the sample surface, while the peak at 420 ps represents the THz wave transmitted through the sample and incident on the metal plate. Several small peaks between 235 ps and 420 ps represent the interfaces between different materials inside the sample. Since the refractive index between different materials inside the sample was small, the peaks of these waveforms were not large.

From the frequency-domain spectrogram, it can be seen that there was a higher signal-to-noise ratio in the frequency range of 0.2–0.6 THz, which can be used for the defect analysis and optical parameter calculation of the sample. Therefore, the subsequent analysis in this study was conducted within this frequency range.

### 3.2. Extraction of Optical Performance Parameters

Assuming the sample was uniform, the sample response signal detected by THz-TDS was stable and did not vary with time [22,23]; the interaction between the THz pulse and the sample was essentially linear, then the transmission transfer function of THz through the sample can be expressed as in Refs. [24,25]:

(1) $$\hat{H}\left(w\right)=\frac{E_{s}\left(w\right)}{E_{a}\left(w\right)}=\frac{{4{\tilde{n}}_{a}\left(w\right)\tilde{n}}_{s}\left(w\right)cos\theta cos\beta }{{\left[{\tilde{n}}_{a}\left(w\right)cos\beta +{\tilde{n}}_{s}\left(w\right)cos\theta \right]}^{2}}\left(exp\left\{\frac{-i\left[x{\tilde{n}}_{s}\left(w\right)-h{\tilde{n}}_{a}\left(w\right)\right]w}{c}\right\}\right)\mathrm{FP}\left(w\right)$$

In the formula, *c* is the speed of light, θ is the incident angle, β is the refracted angle, *x* is the distance of the THz wave transmitting in the sample, *h* is the horizontal distance of THz wave transmitting in the sample, and FP(w) is the Fabry–Perot cavity (F–P) factor, which is the superposition of multiple reflected echoes generated by the F–P effect. In this experiment, the thickness of the foam core was large, which avoided multiple reflections, so we have $\mathrm{FP}\left(w\right)=1$.

${\tilde{n}}_{a}\left(w\right)$ is the complex reflective index of the air:

(2) $${\tilde{n}}_{a}\left(w\right)\approx 1+i0$$

${\tilde{n}}_{s}\left(w\right)$ is the complex reflective index of the sample:

(3) $${\tilde{n}}_{s}\left(w\right)\approx n_{s}\left(w\right)+ik_{s}\left(w\right)$$

In the formula, $n_{s}\left(w\right)$ is the phase characteristic that characterizes the phase of THz wave transmission through the medium, namely the refractive index of the sample; $k_{s}\left(w\right)$ is the extinction coefficient, which represents the absorption of the THz pulse by the material [26].

Under vertical- or small-angle incidence, we have cosθ=cosβ=1. Assuming the sample thickness is *d*, then d=x=h. Considering the complex refractive index of the air ${\tilde{n}}_{a}\left(w\right)\approx 1+i0=1$, the transfer function (1) can be simplified as:

(4) $$\hat{H}\left(w\right)=\frac{{4\tilde{n}}_{s}\left(w\right)}{{\left[1+{\tilde{n}}_{s}\left(w\right)\right]}^{2}}\left(exp\left\{\frac{-i\left[{\tilde{n}}_{s}\left(w\right)-1\right]wd}{c}\right\}\right)$$

Rewriting Equation (4) in the form of the modulus and argument, we have:

(5) $$\hat{H}\left(w\right)=\rho \left(w\right)exp\left[-k_{s}\left(w\right)wd\right]$$

Substituting the extinction coefficient $k_{s}\left(w\right)$ into Equation (5), we can obtain the amplitude–frequency and phase–frequency expressions:

(6) $$\rho \left(w\right)=\frac{4{\left[{n_{s}\left(w\right)}^{2}+{k_{s}\left(w\right)}^{2}\right]}^{1/2}}{{\left[1+n_{s}\left(w\right)\right]}^{2}+{k_{s}\left(w\right)}^{2}}\exp\left[-\frac{k_{s}\left(w\right)wd}{c}\right]$$

(7) $$\phi \left(w\right)=\frac{\left[n_{s}\left(w\right)-1\right]wd}{c}+arctan\left\{\frac{k_{s}\left(w\right)}{n_{s}\left(w\right)\left[n_{s}\left(w\right)+1\right]+{k_{s}\left(w\right)}^{2}}\right\}$$

If the sample absorbs THz waves weakly, $k_{s}\left(w\right)\ll n_{s}\left(w\right)$. And Equations (6) and (7) can be simplified as:

(8) $$\rho \left(w\right)=\frac{4n_{s}\left(w\right)}{{\left[1+n_{s}\left(w\right)\right]}^{2}}\exp\left[-\frac{k_{s}\left(w\right)wd}{c}\right]$$

(9) $$\phi \left(w\right)=\frac{\left[n_{s}\left(w\right)-1\right]wd}{c}$$

From Equations (8) and (9), the refractive index and extinction coefficient of the sample can be determined:

(10) $$n_{s}\left(w\right)=1+\frac{\phi (\;w)\;c}{wd}$$

(11) $$k_{s}\left(w\right)=\frac{c}{wd}ln\left\{\frac{4n_{s}\left(w\right)}{{\rho \left(w\right)\left[1+n_{s}\left(w\right)\right]}^{2}}\right\}$$

By the extinction coefficient, the absorption coefficient can be determined as:

(12) $$\alpha_{s}\left(w\right)=\frac{{2k}_{s}\left(w\right)w}{c}=\frac{2}{d}ln\left\{\frac{4n_{s}\left(w\right)}{{\rho \left(w\right)\left[1+n_{s}\left(w\right)\right]}^{2}}\right\}$$

**Figure 7:**
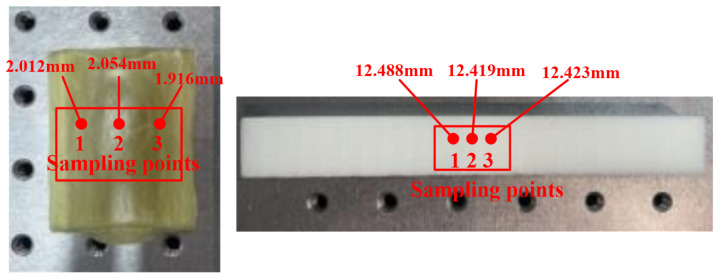
Three sampling points are randomly selected for the samples.

Three sampling points were randomly selected for Sample #3 and Sample #4, and the thickness of each point was measured 5 times to obtain the average value as the actual thickness of that point, as shown in Figure 7.

**Figure 8:**
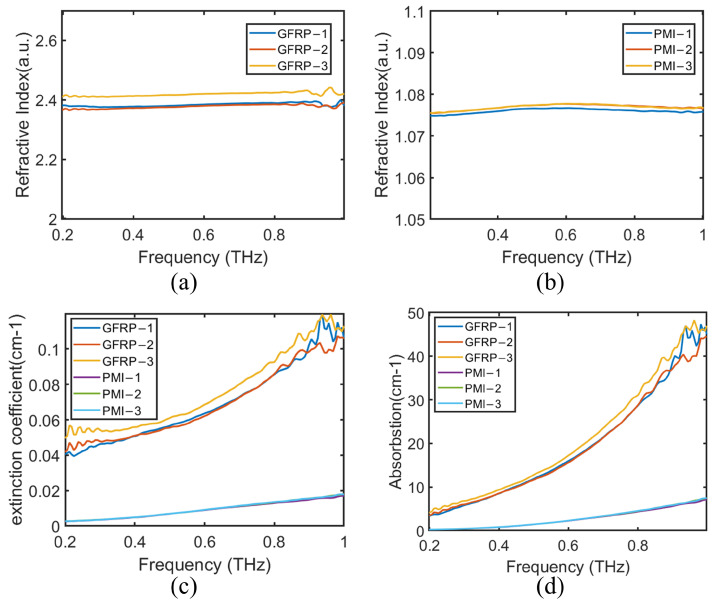
The optical performance parameters of the GFRP panel and PMI foam: (**a**) Refractive index of GFRP panel. (**b**) Refractive index of PMI foam. (**c**) Extinction coefficient. (**d**) Absorption coefficient.

The THz spectra of each sampling point were collected. Based on the detected THz signals and the above formulas, the optical performance parameters of the GFRP panel and PMI foam were calculated, and the relative refractive index, extinction coefficient, and absorption coefficient were also extracted; please see Figure 8.

Due to the thickness differences at the sampling points, the optical parameters of the materials fluctuated within a certain range. The results in Figure 8 show the following: (1) the refractive index of the GFRP laminate was between 2.368 and 2.412, while the refractive index of the PMI foam was between 1.074 and 1.076; (2) the extinction coefficient and absorption coefficient of the GFRP laminate and PMI foam enlarged with the increase of the frequency, and the increase in the GFRP laminate was greater than that in the PMI foam; (3) the extinction coefficient and absorption coefficient of the GFRP laminate were both larger than those of the PMI foam. It can be seen that the optical parameters of GFRP and PMI materials had significant differences and could be well distinguished in the THz band, making them suitable for non-destructive testing.

### 3.3. Principle of Non-Destructive Test

**Figure 9:**
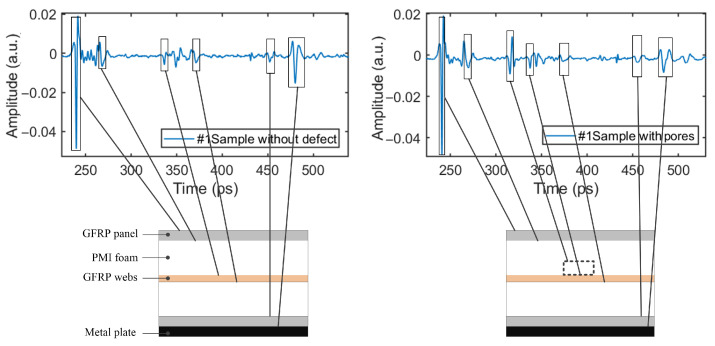
Reflected THz signals at defect and non-defect positions of Sample #1 (electric-field waveform related to time [13]).

If the testing samples contain defects on the surface or inner part, such as inclusions, pores, and cracks, the THz wave incidence on the defect positions would show reflection or scattering, and a varied amplitude and phase. Therefore, the THz signal in the defect area will be significantly different from that in the non-defect area, which forms the theoretical basis for defect identification using THz technology. As shown in Figure 9, by comparing and analyzing the time-domain spectral information of the sample at defect and non-defect positions, defect information can be collected.

**Figure 10:**
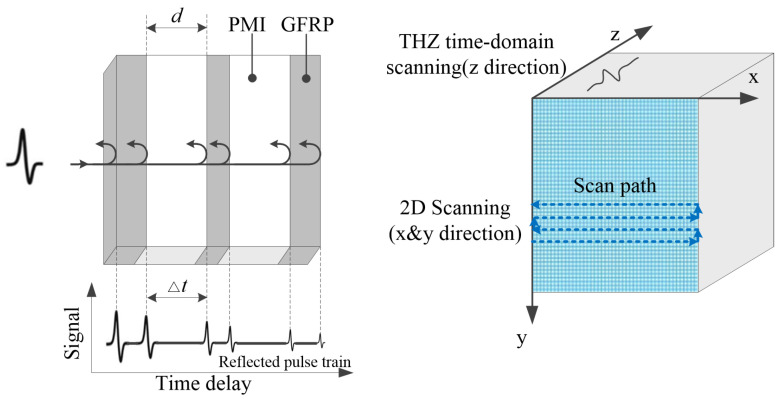
The principle of THz-TDS non-destructive testing.

During the measurement process, the to-be-tested sample was fixed on a two-dimensional electric translation stage, and the position of the sample was changed by moving the stage (with a step size of 0.5 mm) to scan different points of the sample with the THz pulse. Then, the THz signals at different points were reconstructed in three dimensions to obtain the three-dimensional data. Based on the analysis of single-point THz signals, the longitudinal depth information of defects can be obtained. And combining with the mechanical positioning parameters of the two-dimensional scan, the lateral position information of defects can be obtained as well, thus locating the defects. The principle of THz-TDS non-destructive testing is as shown in Figure 10.

### 3.4. Imaging Algorithm

The process of THz non-destructive testing imaging is mainly to compare the THz signals in the defect and non-defect areas and determine the position and size of the defects according to the signal differences, thus generating detection images. Based on existing research, for THz time-domain signals, imaging can be performed using certain information such as the peak amplitude, pulse width, and time-of-flight. The main imaging algorithms include peak-to-peak imaging, maximum-amplitude imaging, minimum-amplitude imaging, pulse-width imaging, time-of-flight imaging, etc. [27,28,29,30].

**Figure 11:**
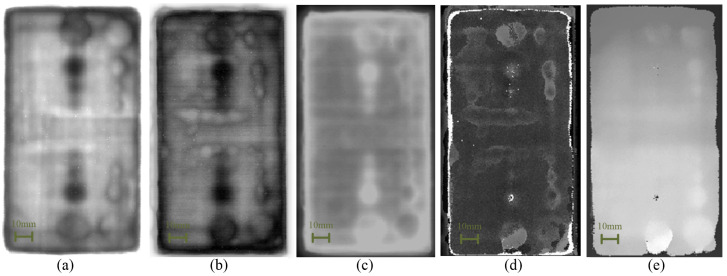
The results generated by by different imaging algorithms: (**a**) Peak-to-peak imaging. (**b**) Maximum-amplitude imaging. (**c**) Minimum-amplitude imaging. (**d**) Pulse-width imaging. (**e**) Time-of-flight imaging.

The pulse data of a certain time-domain segment of Sample #1 were taken to conduct defect imaging by different imaging algorithms, and the results are shown in Figure 11. By comparing the imaging results, it can be observed that the defect information was blurry in the results generated by pulse-width imaging and time-of-flight imaging, which is not conducive to observation and identification. The contrast between the defect and non-defect positions was low in the results generated by minimum-amplitude imaging, which can easily lead to missed detection. And for the results generated by the maximum-amplitude imaging, though it could detect the defect information, it could also be easily affected by noise, forming a pore-like pattern in the non-defect area. The peak-to-peak-imaging algorithm had a better detection effect for the defects in this experiment, so it was used to detect inclusions, pores, debonding, and crack defects.

**Figure 12:**
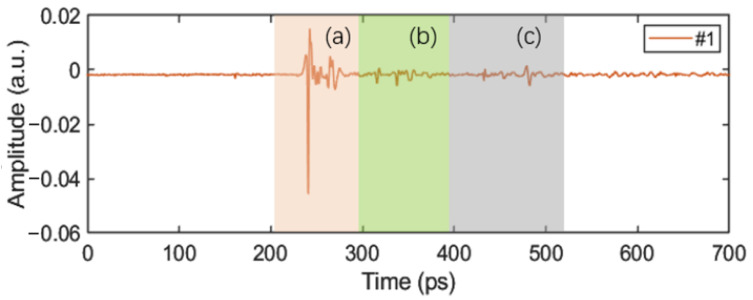
The complete time-domain signal at a certain point of Sample #1: (a) Signal of upper panel. (b) Signal of upper PMI foam core. (c) Signal of lower PMI foam core.

**Figure 13:**
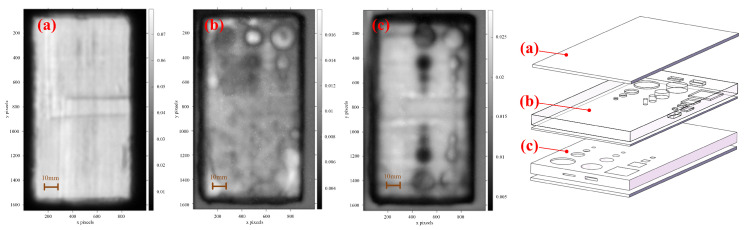
The corresponding relationship between the imaging results and the physical images: (**a**) The upper panel. (**b**) The upper PMI foam core. (**c**) The lower PMI foam core.

Since the defects were distributed at different depths in the sample, in order to detect the defect information at different depth positions, it was necessary to extract the signal data of different pulse periods for processing. Taking Sample #1 as an example, the time-domain signal at a certain point is shown in Figure 12. The time-domain signals at different depths (different color zones) of Figure 12a–c were imaged, and the corresponding relationship between the imaging results and the physical images is shown in Figure 13a–c. Due to the large range of the selected depths, the defect information can be easily masked by other information (waveform changes caused by different material interfaces), resulting in a poor defect-detection effect. To improve the detection effect, the range of selected depths can be narrowed, as shown in Section 4.

### 3.5. Super-Resolution Reconstruction

**Figure 14:**
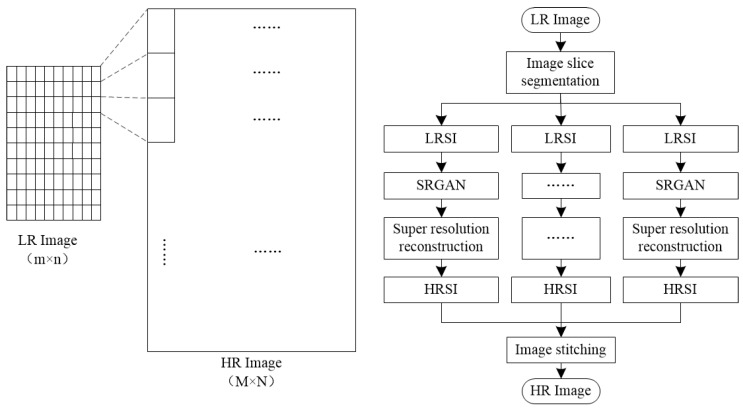
The image slice-segmentation-reconstruction algorithm based on SRGAN.

The existing THz-TDS adopts scanning imaging. Due to the step size restriction of the two-dimensional translation stage, the imaging resolution was only 200 × 330 pixels, which is too low to observe the details of the defects. Therefore, in this paper, an image slice-segmentation-reconstruction algorithm based on SRGAN is proposes—SSSRGAN. As shown in Figure 14, this method can improve the display resolution of THz imaging results in the image post-processing stage and overcome the hardware limitations. First, the original low-resolution (LR) image was divided into several low-resolution sub-images (LRSIs), and then, the SRGAN algorithm [31,32,33] was used to perform super-resolution reconstruction on each LRSI to generate high-resolution sub-images (HRSIs). Finally, the super-resolution reconstructed HRSIs were stitched together to form a complete super-resolution image. Compared with the traditional SRGAN algorithm, this method can also perform multi-threaded parallel computation, thereby improving computational efficiency.

**Figure 15:**
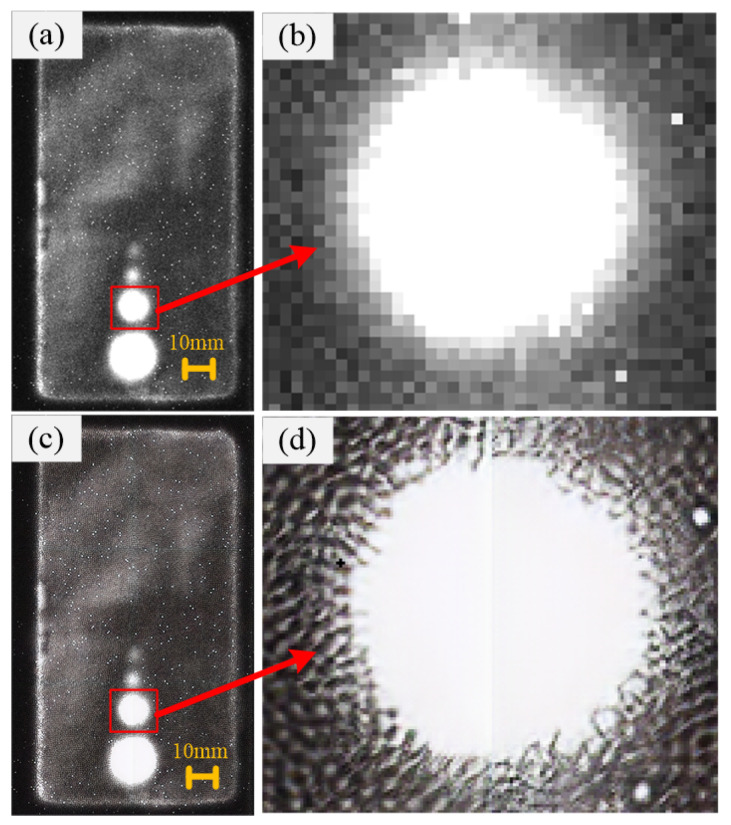
The comparison of the images before and after super-resolution reconstruction: (**a**) Low-resolution original image. (**b**) Locally enlarged image of the low-resolution image. (**c**) Super-resolution reconstructed image. (**d**) Locally enlarged image of the super-resolution reconstructed image.

The comparison of the images before and after super-resolution reconstruction is shown in Figure 15. Figure 15a,b are the low-resolution original image and its locally enlarged image, while Figure 15c,d are the super-resolution reconstructed image and its locally enlarged image. It can be seen that the defect edge contours in the locally enlarged image of the low-resolution image are blurred, while the defect edge contours in the locally enlarged image of the super-resolution reconstructed image are clear. This indicates that the proposed super-resolution reconstruction algorithm in this paper has obviously better effects.

The peak signal-to-noise ratio (PSNR) was taken as the evaluation index to make the comparison among the linear interpolation algorithm, the bicubic interpolation algorithm, and the SSSRGAN algorithm proposed in this paper. The PSNR values of the above three were calculated to be 26.424, 25.643, and 18.557, respectively, indicating that the SSSRGAN algorithm proposed in this paper is superior.

## 4. Results and Discussions

Taking Sample #1 as an example, for the defects of pores, debonding, inclusions, and cracks, the time-domain signal data at the corresponding depth positions were selected and imaged by the peak-to-peak algorithm. Different defects were symmetrically distributed in the upper and lower PMI foam cores, as shown in Figure 1 and Figure 2.

### 4.1. Pore Defect

**Figure 16:**
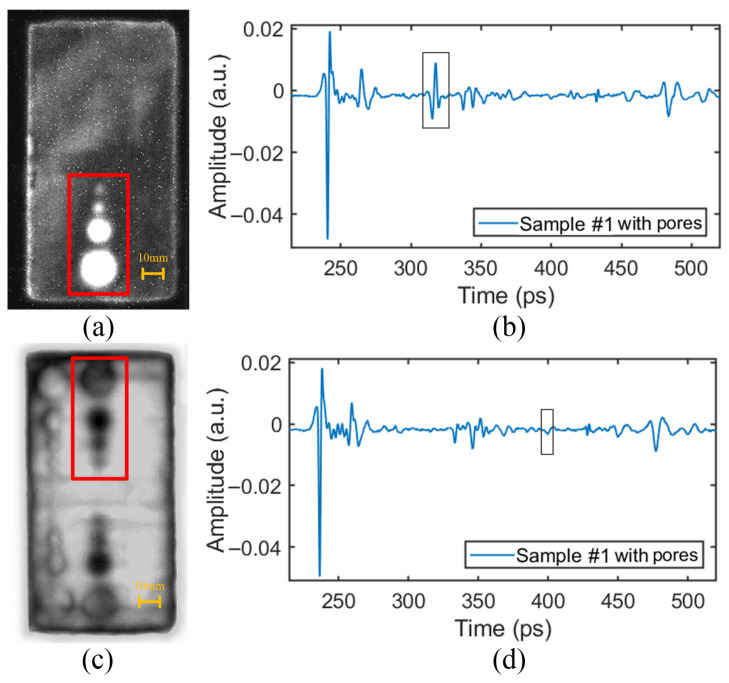
The time-domain signals and relative imaging results of pore defects: (**a**) The detection image of pore defects in the upper PMI foam core. (**b**) The time-domain signal at the position of the pore defect in the upper PMI foam core. (**c**) The detection image of pore defects in the lower PMI foam core. (**d**) The time-domain signal at the position of the pore defect in the lower PMI foam core.

The time-domain signals at the positions of pore defects in the upper and lower PMI foam cores are as shown in Figure 16b,d. Referring to the time-domain waveform at the defect-free position in Figure 9, it can be seen that the pore defect in the upper PMI foam core caused the THz signal to form a significant reflection wave (as shown in Figure 16b. This is because, when the THz wave enters the air (low refractive index) in the pore from the PMI foam (high refractive index), the refractive index difference between the two media is large, resulting in a large peak value of the reflection wave. However, the peak value of the reflection wave corresponding to the pore defect in the lower PMI foam core shown in Figure 16d is much smaller than that in Figure 16b. This is because, when the THz wave passes through the upper PMI foam core and the web, it experiences energy attenuation, which leads to the peak value reduction of the reflection wave.

The pore defect can be detected based on the reflected waveform at the defect locations. The relative imaging results are as shown in Figure 16a,c. The results indicated that the position, size, and contour information of the pores could be clearly detected. And the features of the pore area with only a 3 mm diameter could even be detected.

Comparing Figure 16a,c, it can be found that the defects in the upper PMI foam core were more obvious than those in the lower PMI foam core. This is because the THz wave is attenuated after passing through the upper-layer material, resulting in a smaller difference in the waveform between the positions with and without defects in the lower PMI foam core, and thus, the imaging contrast was weakened.

It should be noted that Figure 16c not only shows the pore defects in the lower PMI foam core, but also shows the defects in the upper layer. This is because the THz wave first passed through the upper PMI foam core before entering the lower PMI foam core. Therefore, when the THz wave reached the defect in the lower PMI foam core, it already contained the defect information in the upper layer material, which was ultimately reflected in the imaging. Here, only the pore defects in the lower PMI foam core need to be focused on.

### 4.2. Debonding Defect

**Figure 17:**
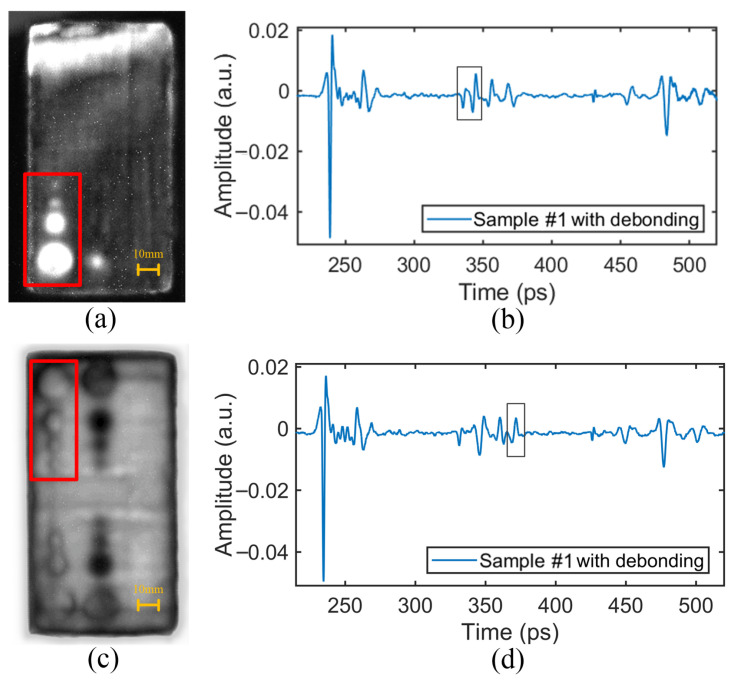
The time-domain signals and relative imaging results of debonding defects: (**a**) The detection image of debonding defects between the upper PMI foam core and the web. (**b**) The time-domain signal at the position of the debonding defect between the upper PMI foam core and the web. (**c**) The detection image of debonding defects between the lower PMI foam core and the web. (**d**) The time-domain signal at the position of the debonding defect between the lower PMI foam core and the web.

The time-domain signals at the positions of debonding defects between the upper and lower PMI foam cores and the web are shown in Figure 17b,d. It can be seen that the debonding defects led to THz signal peaks, which can be used as features to detect the defect information. The data of the pulse period corresponding to the defects were extracted for imaging.

The detection results of the debonding defects are shown in Figure 17a,c. The positions, size, and contour information of the debonding defects could be clearly identified. And the features of the debonding area with only a 3 mm diameter could even be detected.

Comparing Figure 17a,c, it can be found that the debonding defects in the upper PMI foam core were more obvious than those in the lower PMI foam core. This is also because the THz wave was attenuated after passing through the upper PMI foam core and the web, resulting in a lower imaging contrast for the debonding defects between the lower PMI foam core and the web. The brighter area at the upper end of Figure 17a may be caused by the unevenness of the epoxy resin adhesive between the web and the PMI foam core.

### 4.3. Inclusion Defect

The inclusion (square) defect was simulated by inserting paper (with a thickness of 0.053 mm) into the bonding surface between the web and the PMI foam, as shown in Figure 1.

**Figure 18:**
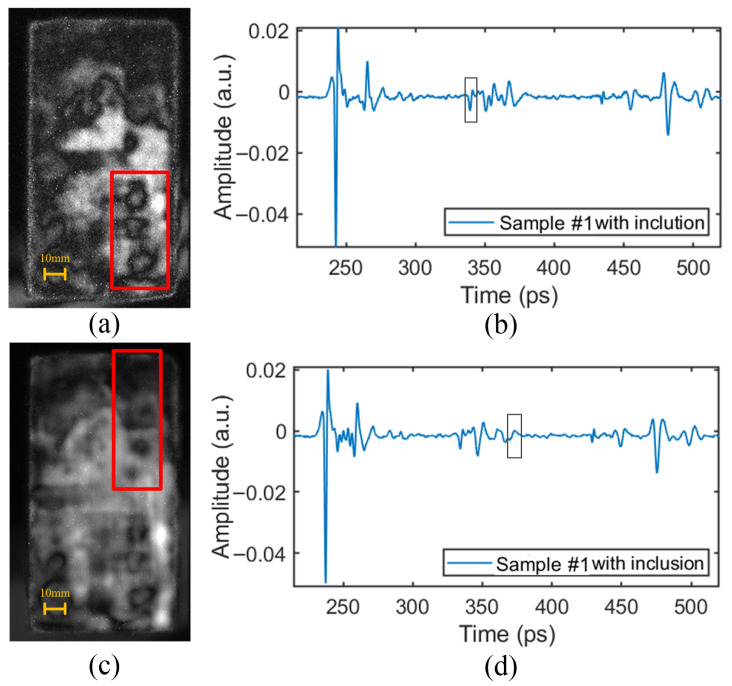
The time-domain signals and relative imaging results of inclusion defects: (**a**) The detection image of inclusion defects between the upper PMI foam core and the web. (**b**) The time-domain signal at the position of the inclusion defect between the upper PMI foam core and the web. (**c**) The detection image of inclusion defects between the lower PMI foam core and the web. (**d**) The time-domain signal at the position of the inclusion defect between the lower PMI foam core and the web.

The time-domain signals at the positions of the inclusion defects between the upper and lower PMI foam cores and the web are shown in Figure 18b,d. Referring to the time-domain waveform at the defect-free position (Figure 9), it can be found that the inclusion defect in the upper layer made the peak value of the waveform increase slightly, while the inclusion defect in the lower layer caused the THz signal to form a small peak, as shown in the boxed area in Figure 18b,d. The signal data corresponding to the pulse period of the defects were extracted for imaging, and the results are shown in Figure 18a,c.

The imaging results showed an unsatisfactory effect of inclusion defect detection. The detected defect features were unclear and could only be used to distinguish the existence of the defect. This is because the paper was too thin (0.053 mm), and its influence on the THz pulse waveform was very weak, resulting in little difference in the features between the positions with and without defects. Moreover, it could also be easily affected by noise interference.

### 4.4. Crack Defect

**Figure 19:**
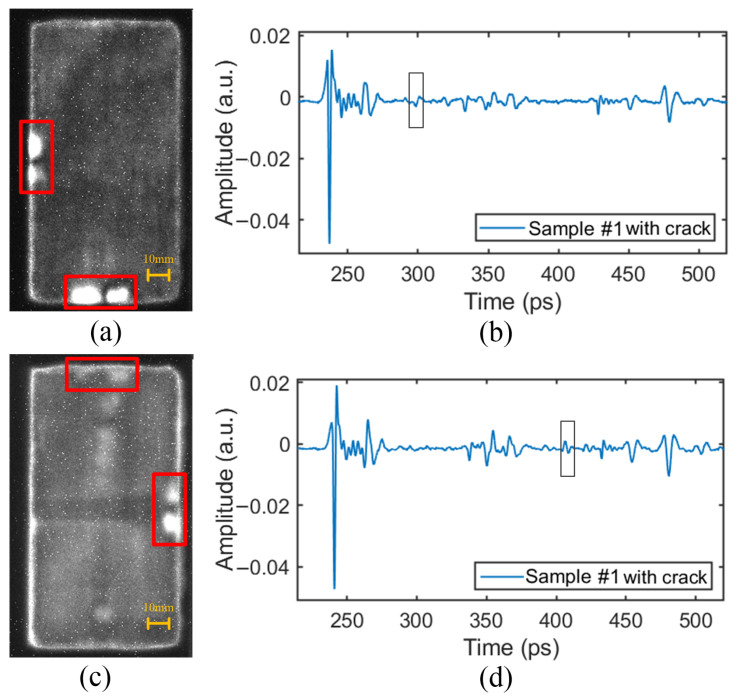
The time-domain signals and relative imaging results of crack defects: (**a**) The detection image of crack defects in the upper PMI foam core. (**b**) The time-domain signal at the defect position in the upper PMI foam core. (**c**) The detection image of crack defects in the lower PMI foam core. (**d**) The time-domain signal at the defect position in the lower PMI foam core.

The time-domain signals at the defect position are shown in Figure 19b,d. The THz wave entered the crack gap from the PMI foam (high refractive index to low refractive index), and then, entered the PMI foam from the crack gap (low refractive index to high refractive index), resulting in the formation of a small wave peak in the THz signal. This characteristic can be used to detect crack defects.

The crack detection results in the upper PMI foam core are shown in Figure 19a. The results indicated that the crack position in the foam core was accurately detected, and the imaging of the 2 mm wide crack was more obvious than that of the 1 mm wide crack. This is because the larger the crack width, the longer the distance that the THz pulse propagated in the crack gap (different reflective index from that of PMI foam). Compared to those propagating entirely in the PMI foam, such pulse transmission would have a more obviously different waveform.

The crack detection results in the lower PMI foam core are shown in Figure 19c. The brightness of the crack defect in the lower PMI foam core was lower. This is also because the THz wave experienced energy attenuation after passing through the upper layer of the material, resulting in weaker imaging contrast. However, the crack could still be detected and located.

Overall, the edge contour of the crack was not very clear. There may be two reasons for this: (1) During the sample preparation process, the resin filled in some of the crack gaps, causing interference; (2) the crack was not completely cut through during the crack processing, and there was still foam present in the uncut corners, resulting in a relatively blurry image.

Sample #2 was only 20 mm, smaller than that of Sample #1. And the THz defect detection could also effectively detect internal defects in this sample, which will not be further elaborated here. In general, THz-TDS can effectively detect pores, cracks, and debonding, showing great promise for non-destructive testing applications in sandwich panels.

As the defect size increased along the propagation direction of THz waves, the distance over which THz waves propagated in the defect region (different reflective index from that of the non-defective region) also increases. Consequently, the temporal waveform differences between the defect and non-defect regions became more pronounced, leading to higher imaging contrast. Moreover, when the defect size was larger perpendicular to the propagation direction of the THz waves, the detected defect area in the imaging results also increased.

The quality and stability of our method were estimated. Calculate the probability *P* that all defects of various sizes and origins were detected. The probability *P* of Sample #1 was 93.75%, and the probability *P* of Sample #2 was 96.88%. The probability *P* primarily depended on the inclusion defects, with smaller sizes being more prone to missed detections.

### 4.5. Calculation of Sample Structure Thickness

By comparing the THz time-domain waveforms of the samples with the reference signal, the time delay Δt of the maximum peak of the time-domain waveform of the reference signal and the sample signal was caused by the refractive index differences existing in the air and the samples. This time delay can be expressed as:

(13) $$\Delta t=\frac{ln_{s}}{c}$$

where *l* is the transmission distance of the THz pulse in the sample. For reflective measurement mode, l=2d, where *d* is the sample thickness. According to the time delay Δt, the estimated formula for the thickness of the tested material can be obtained:

(14) $$d=\frac{c\Delta t}{2n_{s}}$$

where *c* is the speed of light and $n_{s}$ is the refractive index of the tested material. From Figure 8, it can be seen that the refractive index of the GFRP laminate was 2.380 and the refractive index of the PMI foam was 1.075.

The total thickness of Sample #1 was 30 mm, and the total thickness of Sample #2 was 20 mm. The thickness information of the panel, foam core, and web of Sample #1 and Sample #2 was calculated using Equation (14), as shown in Table 2. It can be seen that the maximum error of Sample #1 was 0.870 mm, and the maximum error of Sample #2 was 0.270 mm. The measurement error of Sample #1 was larger than that of Sample #2, because Sample #1 was thicker. When detecting thicker structures, the THz signal was attenuated more and was more susceptible to interference signals, resulting in increased calculation errors.

## 5. Conclusions

To verify the feasibility of using THz technology for detecting defects in fiber-web-reinforced PMI foam sandwich panels, the THz transmission signals of GFRP laminate and PMI foam were firstly extracted, and their refractive index and extinction coefficient were calculated. Then, FWRCSP samples with thicknesses of 30 mm and 20 mm were prepared, and different sizes of pore, debonding, inclusions, and crack defects were pre-made in the samples for detection and imaging. The following conclusions were drawn:

(1) The THz pulse can penetrate and detect defect features in samples with thicknesses of 20 mm and 30 mm, making it suitable for detecting internal defects in fiber-web-reinforced PMI foam sandwich panels.

(2) The frequency-domain spectrum analysis of the samples showed that the 0.2–0.6 THz frequency range had a higher signal-to-noise ratio and could be used for defect analysis of the samples using THz waves in this frequency range.

(3) This study used THz-TDS for defect detection in the FWRCSP for the first time. The results showed that THz-TDS can clearly detect different sizes of pore, debonding, and crack defects, with a minimum detectable size of 3 mm pores and debonding and a minimum detectable crack width of 1 mm. Although the detection effect of the 0.053 mm thick inclusions was poor, the defect features could still be detected.

(4) The defect detection effects of peak-to-peak-imaging, maximum-amplitude-imaging, minimum-amplitude-imaging, pulse-width-imaging, and time-of-flight-imaging algorithms were compared, which verified that the peak-to-peak-imaging algorithm had the best effect for detecting internal defects in the sandwich panel.

(5) To address the low resolution problem of existing scanning THz-TDS imaging, an image slice-segmentation super-resolution reconstruction method (SSSRGAN) was proposed, which maintained clear edge contours even after enlarging low-resolution defect images.

(6) Based on the collected THz time-domain signals, the thicknesses of the panel, foam core, and web of the samples were calculated. The maximum error of Sample #1 was 0.870 mm, and the maximum error of Sample #2 was 0.270 mm, indicating that THz-TDS can be used to measure the structural thickness of sandwich panels, which is of great significance in practical applications.

## Figures and Tables

**Table 1:** The comparison of our work and the other works.

Items	Our Work	Other Works
Detection of object	Thick and complex structure	Thin and simple structure
Imaging algorithm	Different imaging algorithms were compared	Fewer imaging algorithms were compared
Resolution	Super-resolution reconstruction (SSSRGAN)	Low resolution of scanning imaging
Thickness information	Calculating the thickness of thick and complex structures	Mainly detecting the thickness of single media

**Table 2:** The thickness information of the panel, foam core, and web of Sample #1 and Sample #2.

No.	Parts	Measurement/mm	Reference/mm	Error/mm
	Upper panel	1.947	2.000	0.053
	Upper foam core	12.654	12.400	−0.254
#1	Fiber web	1.380	1.200	−0.180
	Lower foam core	11.530	12.400	0.870
	Lower panel	1.750	2.000	0.250
	Upper panel	2.045	2.000	−0.045
	Upper foam core	7.325	7.400	0.075
#2	Fiber web	1.393	1.200	−0.193
	Lower foam core	7.255	7.400	0.145
	Lower panel	1.730	2.000	0.270

## Data Availability

The data presented in this study are available upon request from the corresponding author.
